# Identification and Visualization Textile Fibers by Raman Imaging

**DOI:** 10.3390/ma18071682

**Published:** 2025-04-07

**Authors:** Kaili Liu, Huacai Chen

**Affiliations:** College of Optical and Electronic Technology, China Jiliang University, Hangzhou 310018, China

**Keywords:** textile fibers, Raman spectroscopy

## Abstract

Textile fibers are an essential component of daily necessities and are often used as forensic evidence, making their characterization crucial in forensic science. Different types of textile fibers can be identified using their unique Raman spectral characteristic peaks. In this study, we achieved the visualization of single-component, multi-component, and dyed blended fibers through Raman spectral imaging, demonstrating the spatial distribution of different types of textile fibers within the same area. Furthermore, by merging Raman images of fibers from non-confocal planes, we achieved accurate visual identification, providing more possibilities for characterizing fibers with special morphological features using Raman spectral imaging. In conclusion, Raman spectral imaging enables the successful visualization and identification of different types of fibers.

## 1. Introduction

Textile fibers are generally classified into natural fibers and synthetic fibers [1]. Natural fibers are composed of organic plant or animal tissues (such as cotton and wool) or contain certain minerals. Synthetic fibers are formed by extruding raw fiber materials through spinnerets into air or water, creating filament threads. Their different properties determine their performance in the manufacturing processes of industrially produced clothing and other commercially valuable textile materials. Common synthetic fibers include polyethylene terephthalate (PET), polypropylene (PP), and so on [2]. As a component of textiles, fibers often detach or transfer due to external forces such as daily wear and tear. As one of the most common types of forensic evidence, fiber characterization is crucial in forensic science and can be considered a preliminary step in identifying suspicious materials, which may require further in-depth analysis [3]. It is often necessary to characterize the material composition of textile artifacts in cultural heritage archaeology [4]. Historical textiles were typically made from yarns composed of a single type of fiber, although there were also some blended yarns, such as wool blends (containing more than one type of wool) found in medieval textiles [5]. For the identification and analysis of textile fibers, traditional detection methods include visual inspection, microscopic observation, burning tests, and chemical dissolution [6]. Although visual inspection is quick and convenient, it has significant limitations, as the achievable testing accuracy largely depends on the operator’s expertise in recognizing the appearance of different fibers. Regardless of whether the fiber sample is examined from a longitudinal or cross-sectional perspective, microscopic examination remains the most widely used method [7,8]. However, it has certain limitations, as it can only distinguish broad categories of fibers. The chemical dissolution method requires the use of organic solvents, which not only pose significant environmental hazards but also have negative effects on the health of testing personnel, and improper use may result in dangerous situations [6]. In recent years, the development of spectroscopic techniques has accelerated. As non-destructive methods requiring only a minimal sample for analysis, they have been increasingly applied in the identification and characterization of textile fibers through the integration of various technologies. The most commonly used spectroscopic methods include micro-spectrophotometry (MSP) [9], Raman spectroscopy [10,11], and Fourier transform infrared spectroscopy (FTIR) [12]. Cassista [13] evaluated the capability of MSP in the visible range to distinguish green, red, and blue fibers. While spectral differences among most fibers in each color group were evident, only red fibers could be distinctly separated, whereas indistinguishable fibers shared a common source. Although MSP and infrared spectroscopy can accurately identify textile fibers qualitatively, they require stringent temperature and humidity control and samples must undergo drying pre-treatment. The complex sample preparation process and long detection cycles make these methods unsuitable for rapid analysis.

In contrast, Raman spectroscopy is a rapid and non-destructive technique that requires no sample preparation and has higher spatial resolution. It has been successfully applied in forensic science for analyzing paints [14], pigments [15], drugs [16], explosives [17], and textiles. During Raman spectral analysis, molecular vibrations result in a unique spectral fingerprint, enabling highly specific sample characterization, making Raman spectroscopy a valuable tool for fiber identification [18,19]. Raman imaging, which integrates Raman spectroscopy with imaging technology, allows for precise identification of the polymer composition of textile fibers while minimizing false positives. Additionally, it provides quantitative information on fiber size and distribution, offering higher accuracy and applicability in fiber analysis.

Some studies have already applied Raman imaging technology to fiber detection. Zapata [20] used Raman imaging and multivariate analysis to distinguish four groups of fibers and fabrics (including samples of the same category with different colors and samples of different categories with the same color), thereby testing the capability of Raman imaging for textile fiber analysis for the first time. They concluded that although this study was conducted for forensic fiber examination, it could also be applied in other areas, such as quality control in textile manufacturing, for analyzing textile fibers and garment fabrics.

In this study, the feasibility of Raman spectroscopy was tested and various common textile fibers were identified and visualized using Raman spectroscopy and Raman imaging techniques. The experimental results demonstrated that both single-component and multi-component fibers, as well as dyed blended textile fibers, could be effectively distinguished. Moreover, by employing image logic methods, we achieved accurate visual identification of fibers from different non-confocal planes in cotton–polyester blended fabrics, expanding the application of Raman imaging in characterizing fibers with special morphologies and further enhancing its function in the visual identification of dyed textile fibers.

## 2. Materials and Methods

This study focuses on several common textile fibers and dyed blended fabric fibers, including natural fibers such as cotton, silk, and wool, as well as synthetic fibers such as polyester and polyamide, which typically have diameters ranging from 5 to 30 μm. The selected fabric fibers for analysis are cotton–polyester blended textiles. Polyester fibers are further classified into three types based on processing methods: drawn textured yarn (DTY), fully drawn yarn (FDY), and partially oriented yarn (POY). Unless otherwise specified, samples were used in their original form.

Since Raman spectroscopy requires little to no sample preparation, fiber samples were directly attached to glass microscope slides or the reflective side of aluminum foil covering the slides. The use of aluminum foil is intended to reduce glass interference, but it may introduce indirect contamination risks or artifacts due to the interaction between the foil and the slide, such as reflection effects that could affect the accuracy of spectral data. Before the experiment, it was ensured that the aluminum foil’s surface was smooth, avoiding wrinkles or irregular reflections. A small bundle of fiber samples was placed on a glass slide, with a 5 mm × 5 mm aluminum foil section at the center, reflective side facing up. Both ends of the fiber samples were fixed with fine adhesive to ensure precise positioning and prevent curling.

Raman spectra were recorded in air (~24 °C) using a confocal Raman microscope (XploRA PLUS, HORIBA, Palaiseau, France) equipped with 532 nm and 785 nm laser diodes. The laser power value was 35 mW and the instrument featured software-controlled laser power adjustment, allowing changes from 0.1% to 100% to prevent sample damage or optimize signal intensity. Raman signals were collected using 50× objective and 1200 grooves/mm grating, and the spectral range covered 3000–200 cm^−1^ with a data interval of approximately 3.23 cm^−1^. The 532 nm laser was used for common and undyed textile fiber samples, while the 785 nm laser was selected for dyed blended fabric fibers. The selection criteria for the lasers are illustrated in Figures S1 and S2, showing that different lasers yielded better testing results for the two respective sample types. Excessive laser power could burn the sample, whereas insufficient power resulted in weak signals with unclear characteristic peaks. In single-point Raman spectroscopy, the laser power was set to 10% and all experimental samples were analyzed under these conditions. However, during imaging analysis, prolonged laser exposure led to sample burning, especially in wool fibers, even at 10% power. To minimize signal loss while preventing sample damage, the power was reduced to 7% for imaging analysis. Each experiment recorded a Raman spectrum per pixel, making the total number of spectra dependent on the image resolution. Due to variations in sample surface area and scan size, the image dimensions and total spectral count varied across experiments.

In single-component fiber experiments, wool fibers were scanned over a 65 × 42 μm area with 34 × 22 pixels, while cotton fibers were scanned over a 31 × 26 μm area with 16 × 13 pixels. Multi-component fiber experiments covered a 53 × 25 μm area with 28 × 14 pixels, whereas the cotton–polyester blended fabric was scanned twice over an 88 × 55 μm area with 44 × 28 pixels. High-resolution hyperspectral arrays were generated for each physical position (x/y axes). To maintain image clarity, Raman scan durations were adjusted according to pixel size. A low sampling rate could lead to excessive background noise, resulting in blurred pseudo-color images that failed to resolve chemical composition. Image blurring was also caused by fluorescence background signals. To reduce noise interference, baseline correction and smoothing techniques were applied. For fiber mixture imaging, strong, non-overlapping characteristic peaks were selected and different peak intensities were mapped into distinct pseudo-color images.

## 3. Results

### 3.1. Raman Spectrum of Common Textile Fibers

**Figure 1:**
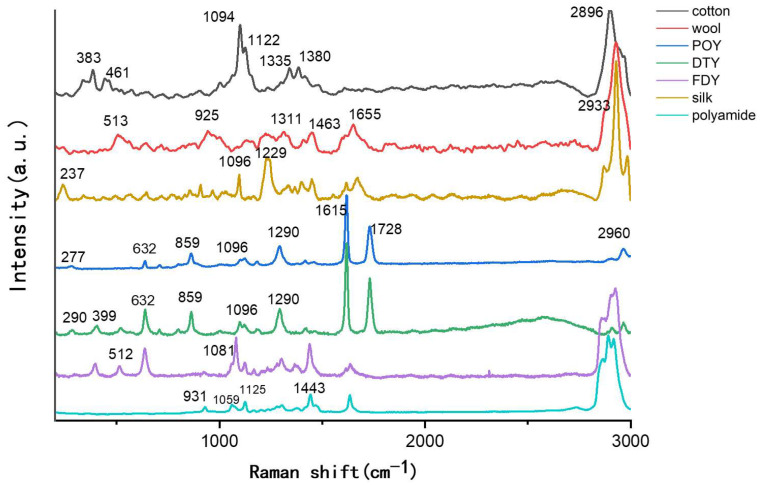
Raman spectrum of textile fibers.

Raman spectra were obtained for several common textile fibers, with baseline correction and smoothing applied to remove spectral background noise. Taking DTY as an example, we conducted ten tests on each fiber bundle; as shown in Figure S3, although the Raman intensity varied, the peak positions remained unchanged. The comparison results are shown in Figure 1, where different colors represent the Raman spectra of different fiber types. Cotton fiber is primarily composed of cellulose and its Raman spectrum exhibits characteristic peaks corresponding to cellulose [20,21,22]: 2896 cm^−1^ (C-H stretching vibration), 1380 cm^−1^ (C-H/CH_2_ bending vibration), 1335 cm^−1^ (O-H bending vibration), 1122 cm^−1^ (asymmetric C-O-C stretching in glycosidic bonds), and 1094 cm^−1^ (symmetric C-O-C stretching in glycosidic bonds). Wool, as a common animal protein fiber, is mainly composed of keratin [23]. Its Raman spectrum [23,24] exhibits strong bands, including 2933 cm^−1^ (C-H stretching vibration), and additional characteristic peaks such as 513 cm^−1^ (S-S bond stretching) [25], and 925 cm^−1^ (C-C skeletal bending). The key distinction between wool and silk fibers lies in the presence of disulfide bonds; as shown in Figure 1, the 513 cm^−1^ peak in the wool Raman spectrum corresponds to the disulfide bond cross-linking provided by cystine [26].

In the Raman spectrum of silk, a sequence of amino acid backbone vibrations appears at 200–500 cm^−1^, including 237 cm^−1^. The dominant bending band of the amide CH_2_ is present at 1229 cm^−1^. For the three types of polyester fibers (DTY, FDY, and POY), the intensity of Raman scattering is stronger than fluorescence, making the peak positions more distinct. In the Raman spectrum of polyester fibers [23], two very strong characteristic bands are present: C-C aromatic ring stretching around 1615 cm^−1^ and carbonyl (C=O) stretching around 1726 cm^−1^. The Raman spectra of these three polyester fiber subtypes also exhibit similar bands at 638 cm^−1^, 1374 cm^−1^, and 2965 cm^−1^. Additionally, FDY lacks the 1726 cm^−1^ (C=O stretching) band, which is present in both POY and DTY, making it distinguishable from the other two. In the textile industry, various solvents are used to provide specific properties to fabrics and their composition and concentrations vary depending on the garment type [27]. Even when the polymer itself is identical, the Raman peaks of these substances can be used to differentiate fiber sources. The characteristic peaks of polyamide fibers include CCO stretching near 931 cm^−1^, two C-C skeletal bands at 1059 cm^−1^ and 1125 cm^−1^, and CH_2_ bending at 1443 cm^−1^, as shown in the last curve in Figure 1.

### 3.2. Raman Imaging of Single-Component Textile Fibers

**Figure 2:**
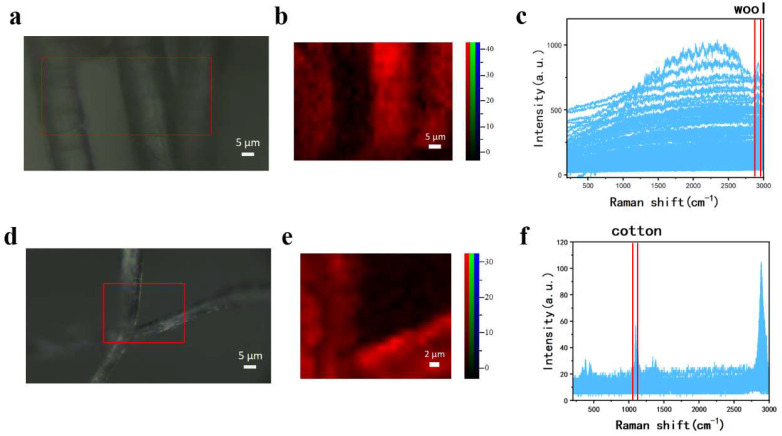
Raman imaging acquisition of wool and cotton fibers. (**a**) Microscopic image of wool fibers and selected scanning area; (**b**) Raman image of wool fibers; (**c**) collected Raman spectra of wool fibers and selected characteristic peak range; (**d**) microscopic image of cotton fibers and selected scanning area; (**e**) Raman image of cotton fibers; and (**f**) collected Raman spectra of cotton fibers and selected typical peak range.

Subsequently, Raman imaging tests were conducted on wool and cotton fibers. Under the microscope, wool fibers appeared as shown in Figure 2a, displaying several fine fibers distributed separately, but their chemical composition could not be determined from the image alone. A 65 × 42 μm region in Figure 2a (marked by a red box) was selected for point-by-point scanning with a step size of 2 μm, obtaining 748 Raman spectra (Figure 2c). The characteristic peak at 2933 cm^−1^ (C-H stretching) was selected for imaging (Figure 2b), which clearly identified the fibers as wool. Additionally, the fiber in the center appeared brighter than the other two, indicating a higher signal-to-noise ratio, likely due to better focusing and positioning of the microscope. This also confirms the advantage of Raman imaging in accurately analyzing fibers even in areas where microscope focus is less clear. Figure 2d–f presents the Raman imaging test of cotton fibers. A 31 × 26 μm region was selected from the microscopic image and scanned with a 2 μm step size, obtaining 208 Raman spectra (Figure 2f). The characteristic peak at 1094 cm^−1^ (symmetric C-O-C stretching in glycosidic bonds) was selected for imaging (Figure 2e). The generated Raman image clearly shows the distribution of cotton fibers, which aligns with the microscopic image. This demonstrates that Raman imaging can effectively visualize the spatial distribution and chemical composition of individual analytes.

### 3.3. Raman Imaging of Multi-Component Textile Fibers

**Figure 3:**
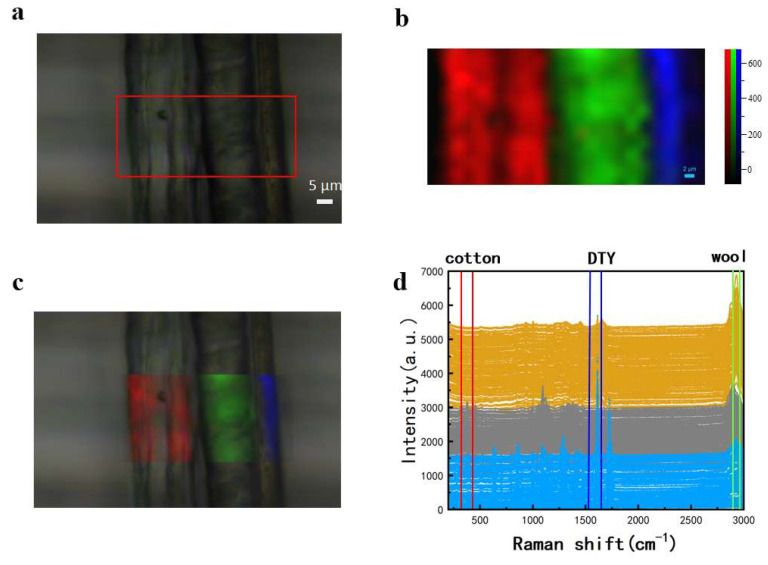
Raman imaging acquisition of DTY, cotton, and wool fibers. (**a**) Microscopic image and selected scanning area; (**b**) Raman image; (**c**) overlay of Raman image and microscopic image; and (**d**) collected Raman spectra of the mixed fibers and selected characteristic peak range.

Further Raman spectral imaging tests were conducted on three mixed fibers: DTY, cotton, and wool, which include two types of natural fibers. The collected data and Raman images are presented in Figure 3. A 53 × 25 μm region was selected for point-by-point scanning with a step size of 2 μm and a total of 392 Raman spectra were obtained, as shown in Figure 3d. It can be observed from the spectrum in Figure 3d that the yellow spectral line, which represents wool fibers, has characteristic peaks at 2933 cm^−1^ and 1655 cm^−1^. The grey has characteristic peaks at 1122 cm^−1^, 1094 cm^−1^, and 461 cm^−1^ which represent cotton fibers. The blue has characteristic peaks at 399 cm^−1^, 1615 cm^−1^, and 1728 cm^−1^ which represent DTY fibers. Due to the proximity of the characteristic peaks of cotton at 1094 cm^−1^ (symmetric C-O-C stretching in glycosidic bonds) and polyester at 1096 cm^−1^ (C-C stretching), three distinct typical peaks, including 461 cm^−1^, 1615 cm^−1^, and 2933 cm^−1^, were selected for imaging. The resulting pseudo-color image (Figure 3b) provides a clear differentiation among the three fibers. The image assigns distinct colors to each fiber type, where the colors represent varying Raman signal intensities. The cotton, wool, and DTY fibers represented by red, green, and blue in Figure 3c correspond well with the microscopic image, confirming the effectiveness of Raman imaging for visual identification of natural fibers (cotton and wool). Additionally, the point-by-point imaging capability of Raman spectroscopy proves to be advantageous in the visualization and analysis of fine mixed fibers.

### 3.4. Raman Imaging of Dyed Blended Textile Fibers

For dyed textiles, dyes significantly interfere with fiber identification. This study investigates various aspects of Raman spectroscopy, including the effect of different laser wavelengths on the spectra of dyed fibers, to determine the optimal laser wavelength and analysis conditions for blended textile fiber identification. A light blue cotton–polyester blended fabric was selected and several fiber clusters were randomly extracted and placed on clean aluminum foil, which was then fixed onto a glass slide. By comparing different excitation wavelengths, it was found that the 785 nm laser produced more distinct Raman characteristic peaks (Figure S2). Thus, the 785 nm laser was used for analyzing dyed blended textile fibers, and it is recommended that longer-wavelength (near-infrared) lasers be used for dyed textiles to mitigate fluorescence interference.

**Figure 4:**
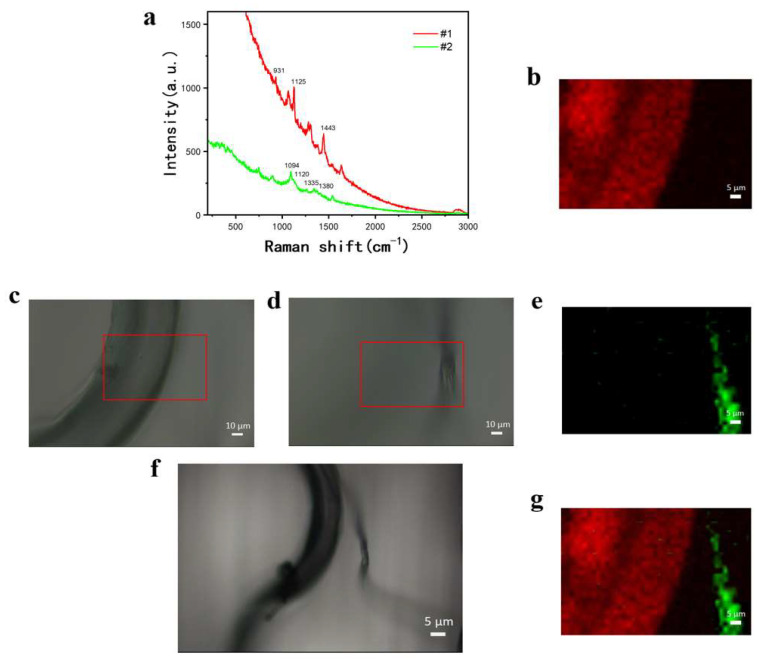
Raman imaging of dyed cotton–polyester blended textile fibers. (**a**) Characteristic Raman spectra collected from different focal planes; (**b**) Raman image (generated by scanning the area selected in (**c**)); (**c**,**d**) microscopic images at 50× magnification with the same x/y coordinates but different focal planes; (**e**) Raman image (generated by scanning the area selected in (**d**)); (**f**) Microscopic image at 10× magnification; and (**g**) merged image from (**b**) and (**e**) using logical OR.

Under 10× magnification, two small fibers located on the outer edge of the primary fiber group were identified as scanning targets (Figure 4f). The scan was then conducted under 50× magnification. However, because the two fibers were not on the same focal plane, it was impossible to focus on both simultaneously, which is a common challenge when scanning textile fibers in dyed fabrics. During the experiment, x/y positioning was controlled via the scanning stage, while z-axis movement was adjusted through focusing. Due to the nature of confocal Raman spectroscopy, signals are collected only within the same focal plane. In a single x/y coordinate scan, the focus remains unchanged, making it impossible to simultaneously scan samples at different focal planes. Consequently, when generating characteristic peak spectral images, only the samples within the current focal plane are displayed. However, textile fibers are inherently interwoven, and it is often difficult to simultaneously focus on different fibers during identification. To address this issue, a multi-plane imaging approach was implemented, where fibers at different focal planes were scanned separately and then merged using an image logic combination method [28]. An 88 × 55 μm region was scanned twice at a 2 μm step size, collecting Raman spectra from different focal planes. The characteristic spectra collected from the first focal plane (#1, Figure 4c) and second focal plane (#2, Figure 4d) were recorded separately. It can be observed from the spectrum in Figure 4a that the green spectral line, which represents cotton fibers, has characteristic peaks at 1120 cm^−1^, 1094 cm^−1^, 1335 cm^−1^, and 1380 cm^−1^. The red has characteristic peaks at 931 cm^−1^, 1125 cm^−1^, and 1443 cm^−1^ and represents polyamide fibers. By adjusting the z position, the physical distance was modified to scan the same x/y coordinates at two different depths, generating two separate Raman spectral matrices. Raman imaging was performed for each plane. The two microscopic images of different focal planes are shown in Figure 4c,d. The Raman imaging result from Figure 4c is shown in Figure 4b, where 1443 cm^−1^ (CH_2_ bending) from polyamide and 1094 cm^−1^ (symmetric C-O-C stretching in glycosidic bonds) from cotton were used for imaging. The Raman imaging result from Figure 4d is presented in Figure 4e, demonstrating a strong correlation between the microscopic and Raman images, confirming the effectiveness of multi-focal Raman imaging in textile fiber identification.

For image merging, ImageJ software 1.52i was used for analysis based on the image logic method. To simultaneously display the Raman imaging results from different focal planes, the images were processed and merged using the logical OR operator. The specific implementation process included the following steps: The original Raman image was opened using the software, which defaulted to the RGB format. To simultaneously display the Raman imaging effects of two scans at different focal planes, the logical OR calculator was used to process and merge the images. The resulting merged image is shown in Figure 4g. In this image, not only do the shape and size correspond to the microscopic image, but a clear visualization of different material compositions is provided, allowing cotton and polyamide fibers to be identified and distinctly displayed. Figure 4g shows the final merged image, where the fiber shapes and sizes correspond to the microscopic images while also providing differentiated material composition visualization.

However, Figure 4g appears blurry. The primary reason is that, although the sample mainly consists of cotton, fibers in actual textiles are often dyed for practical applications, and these dyes can complicate Raman analysis by introducing fluorescence background interference, as shown in the spectral in Figure 4a. Despite the presence of high-intensity fluorescence background interference, the Raman image was still able to display the fiber types and their spatial distribution.

## 4. Conclusions

This study successfully achieved the visual identification of single-component, multi-component, and dyed blended textile fibers using Raman imaging technology. By applying Raman spectral for imaging, pseudo-color images were generated to display the spatial distribution of different fiber components. Additionally, image logic methods were employed to merge Raman imaging results from different focal planes, enhancing the ability of Raman imaging to characterize fibers with complex morphologies.

However, this study has some limitations, including the selection of samples that only contained a few common types of fibers and the testing of only the same type of dyed fiber. Additional, when identifying dyed textile samples, the influence of dyes must be considered. The influence of dyes on fluorescence is acknowledged in this study; however, the extent of this interference, such as the signal-to-noise ratio or penetration depth, has not been quantitatively analyzed. Therefore, developing more efficient methods for the effective visualization of dyed blended fibers remains an important direction for future research.

## Data Availability

The original contributions presented in this study are included in the article. Further inquiries can be directed to the corresponding author.

## Supplementary Materials

The following supporting information can be downloaded at: https://www.mdpi.com/article/10.3390/ma18071682/s1, Figure S1: Raman spectra of undyed fibers with different lasers; Figure S2: Comparison of Raman signal acquisition effects of two lasers on dyed fibers. Figure S3. Raman spectra of DTY fibers tested ten times in same condition.
