# Supplementary material for: Identification and Visualization Textile Fibers by Raman Imaging
Source: Materials (Basel). 2025 Apr 7;18(7):1682. doi: 10.3390/ma18071682 (PMC11990539; doi:10.3390/ma18071682)
Supplement: Supplementary file 1 [file materials-18-01682-s001.zip › materials-3558910-supplementary.pdf]

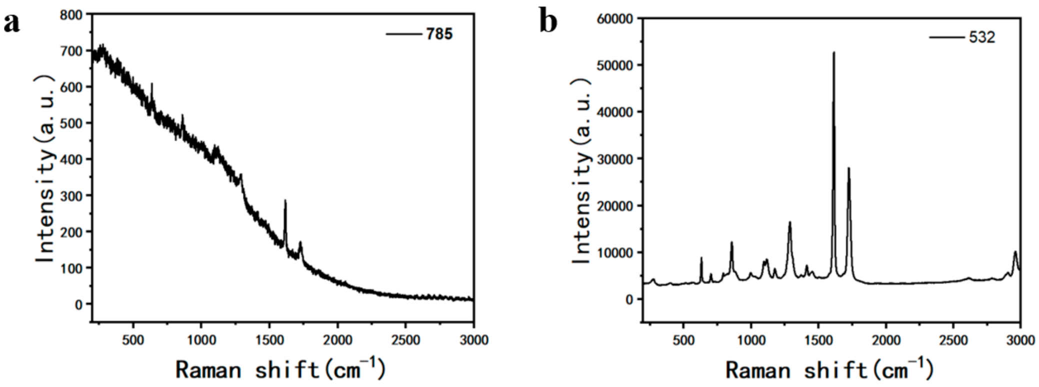

Figure S1. Raman spectra of undyed fibers with different laser. (a) 785 nm; (b) 532 nm.

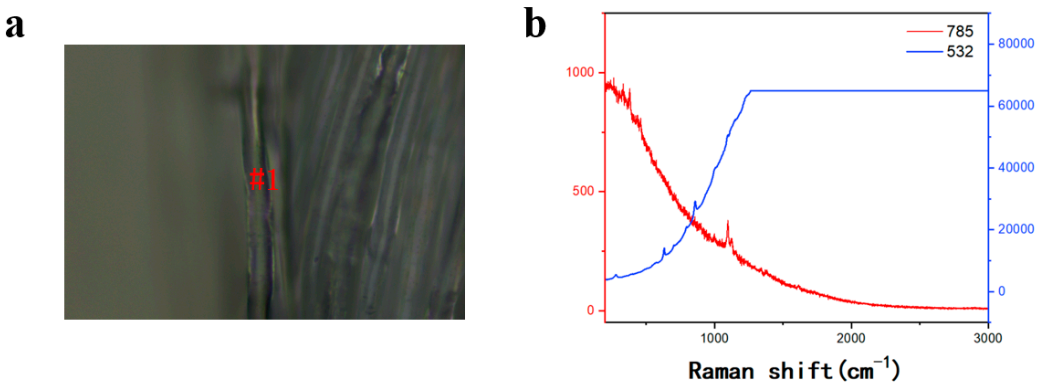

Figure S2. Comparison of Raman signal acquisition effects of two lasers on dyed fibers: (a) microscopic image and the selected spectral acquisition location; (b) Spectra acquired by the two lasers.

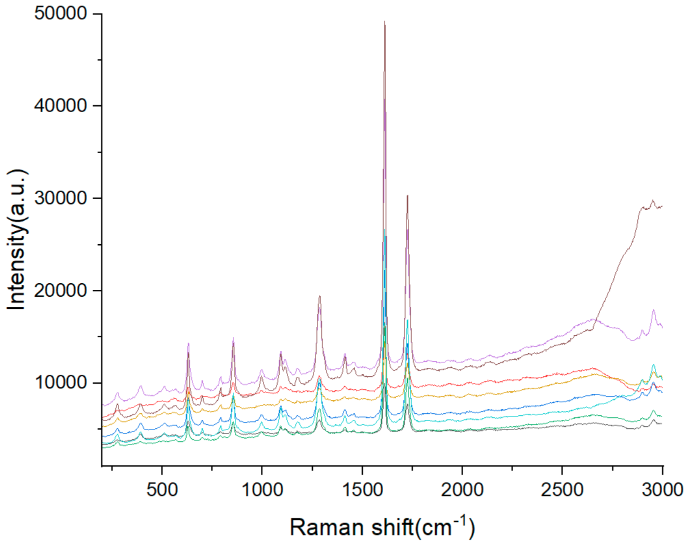

Figure S3. Raman spectra of DTY fibers tested ten times in same condition.
